# The Immunomodulatory Effect of Bone-Marrow Mesenchymal Stem Cells on Expression of TLR3 and TLR9 in Mice Dendritic Cells

**Published:** 2017-02-01

**Authors:** L. Sadeghi, M. H. Karimi, E. Kamali-Sarvestani, N. Azarpira, M. Shariati

**Affiliations:** 1 *Department of Biology, Science and Research Branch, Islamic Azad University, Fars, Iran*; 2 *Department of Biology, Faculty of Science, Shiraz Branch, Islamic Azad University, Shiraz, Iran*; 3 *Transplant Research Center, Shiraz University of Medical Sciences, Shiraz, Iran*; 4 *Autoimmune Disease Research Center, Shiraz University of Medical Sciences, Shiraz, Iran*; 5 *Department of Biology, Islamic Azad University, Kazerun Branch, Kazerun, Iran*

**Keywords:** Mesenchymal stromal cells, Dendritic cell, Antigen-presenting cells, Toll-like receptors, Immunomodulation, Receptors, pattern recognition, Pathogen-associated molecular pattern molecules

## Abstract

**Background:**

Mesenchymal stem cells (MSCs) are multipotent cells with immunomodulatory effect on immune cells including dendritic cells (DCs). DCs are the most potent antigen-presenting cells (APC). MSCs have been found to modulate both differentiation and function of DCs. DCs express a broad range of Toll-like receptors (TLR), which play a critical role in DCs maturation and function.

**Objective:**

To evaluate expression level of TLR3 and TLR9 transcripts in DCs following treatment with MSCs supernatant.

**Methods:**

MSCs and DCs were derived from adult BALB/c mice bone marrow and spleen, respectively. MSCs supernatant was harvested following 24, 48, and 72 hours. Isolated DCs were treated with MSCs supernatant and incubated for 24 and 48 hours. TLR3 and TLR9 transcript levels were evaluated using real-time PCR.

**Results:**

The results showed that 48 and 72 hours MSCs supernatant significantly decreased the expression of TLR3 in DCs following 24 and 48 hours incubation in comparison with untreated cells (p<0.01). Moreover, 48 hours of treatment with 24, 48 and 72 hours MSCs supernatant significantly decreased TLR9 transcript level (p<0.05).

**Conclusion:**

TLR3 and TLR9 mRNA expression decreases in DCs after incubation with MSCs culture supernatant. This confirms the immunomodulatory role of MSCs in cell-base therapy.

## INTRODUCTION

Toll-like receptors (TLRs) are a family of pattern-recognition receptors (PRRs) that recognize conserved structures among microbial species, known as pathogen-associated molecular patterns (PAMPs), and are expressed by different immune cells such as dendritic cells (DCs), T cells, B cells and macrophages [1, 2]. So far, 13 types of mouse TLRs have been identified. TLRs 1, 2, 4, 5, 6, and 11 are expressed on the cell surface, whereas TLRs 3, 7, 8, and 9 are localized in several intracellular organelles including endosomes [2]. Although, TLRs were initially identified as innate immune mediators, recently, several studies have indicated TLR contribution in adaptive immune response. TLR signaling causes up-regulation of MHCII and co-stimulatory molecules on DCs and other antigen-presenting cells (APC) to cause inflammation and antigen-specific T cell responses [3, 4]. Furthermore, TLRs activation induces secretion of soluble factors promoting maturation and differentiation of immune cells such as DCs, which play a critical role in the adaptive immune response [5, 6]. Endosomal TLRs including TLR3 and TLR9 recognize nucleic acid-based PAMPs and provoke immune responses against pathogen-derived nucleic acids [2]. Stimulation of DCs through TLR3 induces production of remarkable pro-inflammatory cytokines, which leads to a systemic inflammatory response [7]. Moreover, TLR9, which recognizes CpG motifs in DNA, has been shown to promote both maturation and antigen presentation by DCs [8]. According to inflammatory properties of TLR3 and TLR9, modulation of TLRs expression and their signaling pathways might be a promising therapeutic approach for treatment of multiple inflammatory conditions, including various autoimmune diseases, atherosclerosis, type 2 diabetes, and osteoarthritis [9].

Bone-marrow derived mesenchymal stem cells (MSCs) are pluripotent stem cells that can differentiate into osteoblastic, adipocytic and chondrocytic lineages in certain condition [10]. MSCs have been found to modulate the immune responses by DCs, attenuate inflammation and repair damaged tissues either by cell-cell contact or secretory proteins including IL-10, IDO, and PGE-2 [11, 12]. A number of recent studies have aimed at the influence of MSCs on DCs function. For instance, MSCs supernatants inhibits maturation of DCs and down-regulates B7 and MHCII, causing induction of tolerogenic DCs (tol-DC), which expresses high level of IL-10 [13, 14]. However, the molecular mechanisms by which MSCs modulate DCs functions remain to be investigated. For example, murine MSCs inhibit DCs activation through TLR4, resulting in inconsiderable inflammatory cytokines production, and inhibiting their migration to the lymph nodes and antigen presentation to T cells [15]. Moreover, MSCs express functional TLRs that can regulate MSCs proliferation and differentiation [16].

Considering TLRs critical roles in DCs maturation and function, as well as MSC capacity to modulate DCs, we postulated that MSCs might regulate immune responses through regulation of TLRs expression. Therefore, expression levels of TLR3 and TLR9 transcripts in mouse DCs treated with MSCs supernatant were assessed.

## MATERIALS AND METHODS

**Mice**

Five- to six-week-old BALB/c and C57BL/6 mice were purchased from Razi Institute (Shiraz, Iran). All mice were inbred and maintained under specific pathogen-free and standard conditions. 

Isolation and Culture of Bone-marrow-derived MSCs

Bone marrow cells were flashed out from femur and tibia bones of BALB/c mice. Isolated cells were cultured in 25 cm^2^ flasks in low glucose Dulbecco modified Eagle medium supplemented with 10% heat-activated fetal calf serum, sodium pyruvate (1%), glutamine (1%), and 100 μg/mL penicillin-streptomycin in CO_2_ incubator at 37 °C. After 24 hrs, non-adherent cells were removed and adherent cells were trypsinized and passaged as described previously [17]. In passage 6, 24, 48, and 72 hrs after trypsinization, supernatants of cells were collected, as MSCs conditioned media, and stored at 20 °C. Purity of the MSCs was determined by flowcytometry using PE-labeled anti-SCA-1, anti-CD45, anti-CD44 and fluorescein-labeled anti-CD34 antibodies. 

***In Vitro*** **Multilineage Differentiation Analysis**

Adipogenesis and osteogenesis of BM-MSCs were assayed in the appropriate induction media. The differentiation phenotype was documented using Oil red O staining for adipocytes and Alizarin red staining for osteocytes.

**Isolation of DCs**

Gradient media, Nycodenz (Axis Shields, Norway) and magnetic-activated cell sorting were used to isolate splenic DCs as described previously [18]. Briefly, mouse spleens were chopped and digested with 1 mg/mL collagenase D (Roche, Germany) and 2 µg/mL DNase (Roche, Germany), and then meshed with 0.2 µm sieves. Cells were washed with RPMI 1640 culture medium (Sigma, St. Louis, MO, and USA) containing 5 mM EDTA. Cell pellets were re-suspended in RPMI 1640 supplemented with 10% fetal calf serum and 5 mM EDTA. The cell suspension was layered on Nycodenz 12.5% (w/v) (d=1.068) and centrifuged at 1800 rpm at 4 °C for 20 min. The interface layer was collected and purified by anti-CD11c micro-magnetic beads (Miltenyi Biotec, Germany) [18]. The purity was determined based on percentage of CD11c expressing cell population using flowcytometry (BD biosciences, USA).

**Primer Design**

TLR3 (NM_126166.4), TLR9 (NM_031178.2) and β-actin (NM_007393.3) specific primers were designed by NCBI primer design software (Biosoft, San Diego, CA, USA). The thermodynamic parameters and secondary structures were determined using mfold software. The primer sequences for TLR3, TLR9 and β-actin were as follows: 

TLR3 F: 5’-GGGCAAGAACTCACAGGCCAGG-3’

TLR3 R: 5’-AAGGGCCACCCTTCGGAGCA-3’

TLR9 F: 5’-CCTCCACGCATGAGGCCCTG-3’

TLR9 R: 5’-CGGCTGCCGACTTGTCCTT-3’

β-actin F: 5’-ATCTACGAGGGCTATGCTCTCC-3’

β-actin R: 5’-AGCCTCGGTCAGGATCTTCAT-3’

**RNA Isolation and Real-time PCR**

Isolated splenic DCs were seeded in 6-well plates and treated with conditioned media obtained from 24, 48, and 72 hrs MSCs supernatant of the 6^th^ passage. The treated DCs were incubated for 24 and 48 hrs; then,TLR3 and TLR9 expressions were evaluated. The cells without treatment were considered “negative controls.” Total RNA was isolated from treated and untreated cells according to the manufacturer’s instruction in three independent experiments using RNX-plus^™^ reagent (CinnaGen, Tehran, Iran). Isolated RNA (1 µg) was reverse-transcribed with random hexamers and oligo d (T) using Vivantis two-steps reverse transcriptase (RT)-PCR kit (Malaysia). Real-time PCR was performed using Takara SYBR Premix Ex taq II in duplicate. The mRNA levels of TLR3 and TLR9 were compared to β-actin mRNA level, as an internal control, by ABI prism 7500 system. Relative gene expression was calculated using ∆∆CT method and β-actin was used as a housekeeping gene for normalization. Finally, expression of each target gene in comparison with reference gene was calculated using 2^∆∆CT^ formula. Reaction with water instead of cDNA template was considered “a non-template control.” Real-time PCR conditions included 95 °C for 2 min as the initial denaturation phase, and 40 cycles of 95°C for 30 sec and 64 °C for 20 sec as elongation. Untreated cells were also considered “negative control.”

**Ethical Considerations**

All of the animal care and experimental procedures were approved by the animal Ethical Committee of Shiraz University of Medical Sciences.

**Statistics Analysis**

Data collected in three independent experiments, were presented as mean±SD. Difference between means of the studied groups was analyzed by one-way ANOVA, followed by Tukey’s HSD, as the *post hoc* test, using Graph Pad Prism 5 software (Graph-Pad Software Inc, San Diego, CA, USA). A p value <0.05 was considered statistically significant.

## RESULTS

**DCs and MSCs Characterization**

**Figure 1:**
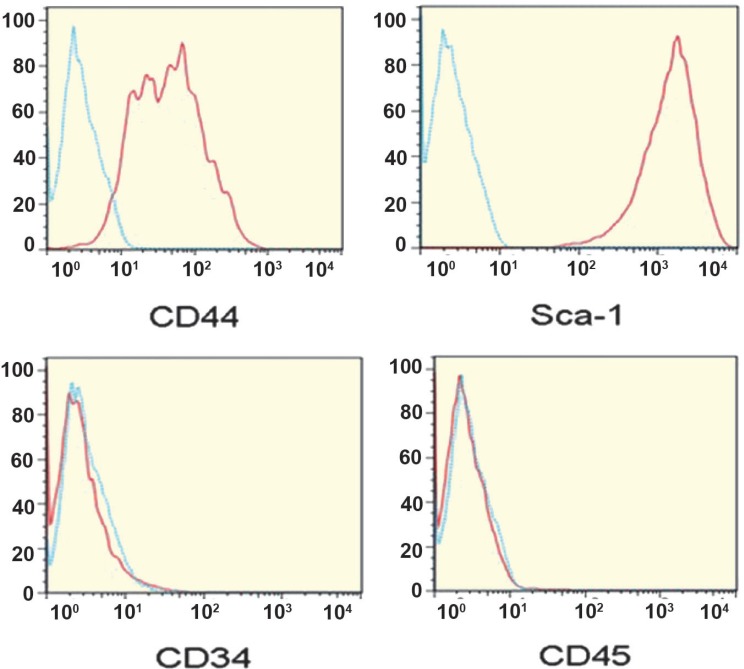
Purity of MSCs was determined using PE-labeled anti-SCA-1, CD44, CD45 and fluorescein-labeled antibodies labeled anti-CD34 antibodies. Almost 95% of cells were positive for CD44 and SCA-1and negative for CD34 and CD45 in passage 5 (solid lines) in comparison with cells stained with isotype control antibodies (dash lines). The results are representative of three independent experiments

**Figure 2:**
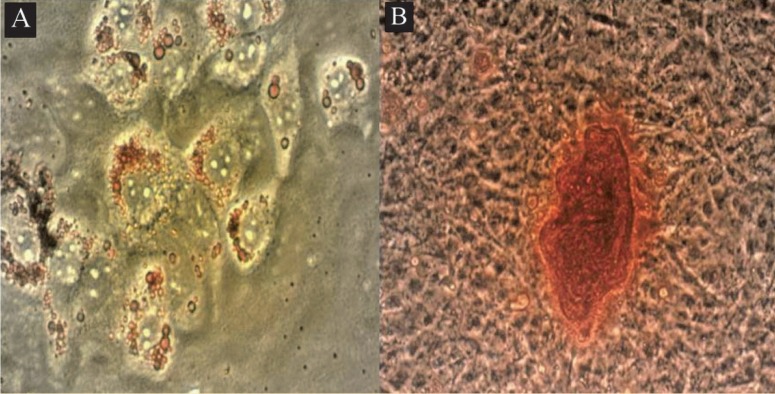
Differentiation of bone-marrow-derived MSCs. A) Adipogenic differentiation of MSCs examined by Oil red O staining for intracellular lipid vacuoles (×100). B) Osteogenic differentiation of MSCs examined by Alizarin red staining (×100

**Figure 3:**
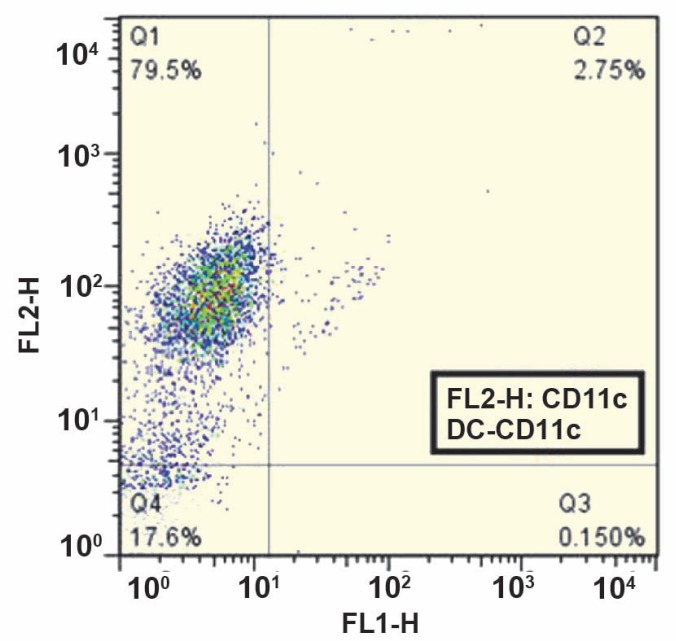
The purity of DCs isolated from spleen. CD11c expression was determined using PE conjugated CD11c antibody. Positive cell population was considered “purified DCs.” The result is representative of three independent experiments

Flowcytometric analysis of MSCs showed that the purity of MSCs was more than 95% based on a pre-dominant population of CD44^+^, Sca-1^+^, and CD34^–^, CD45^–^ cells in passage 5 (Fig 1). Microscopic analysis of MSCs under inductive conditions showed *in vitro* competence of MSCs to differentiate into adipogenic and osteogenic lineages as confirmed by Oil red O staining (Fig 2A), and Alizarin red staining (Fig 2B), respectively. More than 75% of viable cells were positive for CD11c, a DC-specific antigen (Fig 3).

**TLR3 and TLR9 Gene Expression**

**Figure 4:**
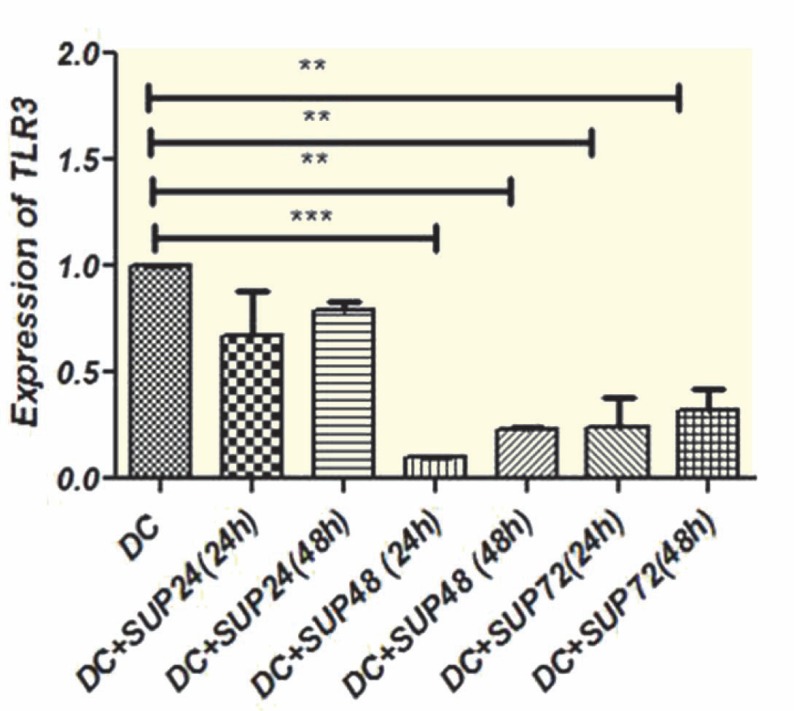
Expression of TLR3 transcript in DCs treated with MSCs supernatant. DCs were treated with MSCs supernatant collected at 24 h, 48 h, and 72 h intervals, and the mRNA level of TLR3 was measured following 24 h and 48 h treatments. 24 h and 48 h treatment with 48 h and 72 h MSCs conditioned media caused significant down-regulation of TLR3 expression compared to untreated cells. Data represent mean±SD of three independent experiments. (**p<0.01, ***p<0.001) (Sup: MSCs supernatant

**Figure 5:**
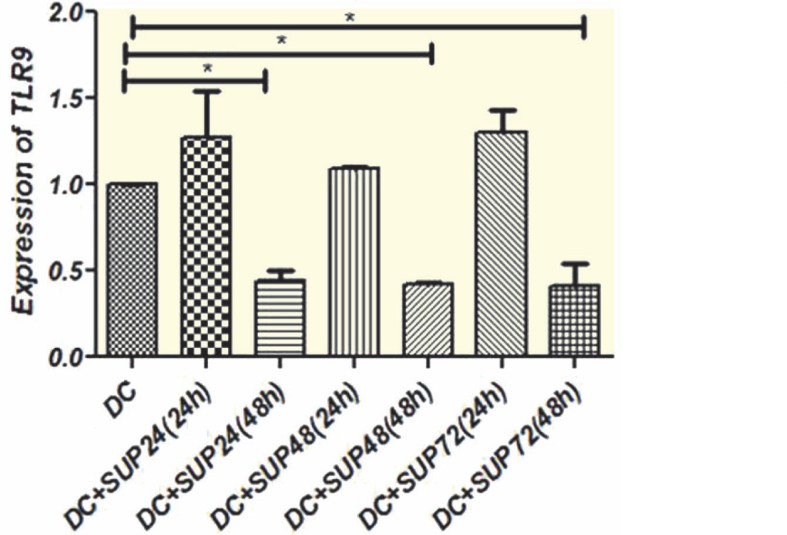
Expression of TLR9 transcript in DCs treated with MSCs supernatant. DCs were treated with MSCs supernatant, collected at 24 h, 48 h and 72 h intervals, and mRNA level of TLR9 was measured after 24 h and 48 h treatment. TLR9 expression was decreased significantly with 24 h, 48 h, and 72 h MSCs supernatant after 48 h incubation compared to untreated cells. Data represent mean±SD of three independent experiments. (*p<0.05) (Sup: Supernatant, h: hours

TLR3 and TLR9 transcript levels in DCs treated with MSCs supernatant were compared to untreated cells following 24 and 48 hrs; 24 and 48 hrs treatments of DCs with 48 and 72 hrs conditioned media of MSCs resulted in down-regulation of TLR3 transcripts in comparison with untreated cells (p<0.01) (Fig 4). Moreover, 24 and 48 hrs treatment with 48 and 72 hrs conditioned media of MSCs caused significant (p<0.05) decrease in mean±SD TLR3 transcript (0.099±0.00 and 0.24±0.14, respectively) compared to 24 hrs conditioned media of MSCs (0.67±0.21) (Fig 4). TLR9 expression was also decreased due to treatment with MSCs supernatant. DCs treatment with 24, 48, and 72 hrs conditioned media of MSCs for 48 hrs, resulted in significant (p<0.05) down-regulation of mean±SD TLR9 mRNA level (0.44±0.06, 0.42±0.01, and 0.41±0.12, respectively) (Fig 5). TLR9 transcript levels of DCs treated with MSCs (24, 48, and 72 hrs MSCs supernatant) conditioned media for 48 hrs were significantly down-regulated comparing to 24 hrs treatment.

## DISCUSSION

Stimulation through TLRs provides a vital signaling for DCs activation in response to antigenic stimuli [19, 20]. TLR3 and TLR9 functional expressions on DCs have been reported earlier [21]. Activated and matured DCs by TLRs are able to present pathogen-derived peptides to T cells, inducing T cell activation, and differentiation and cell-mediated adaptive immune responses [20]. Some studies demonstrated the importance of TLR9 signaling for Th1-mediated immune responses [22]. Absence of TLR9 inhibits the development of protective Th1 response transiently [23, 24]. TLRs also trigger T cell stimulatory function and attenuate the differentiation into T-regulatory cells [25]. It has been shown that MSCs, as the immunomodulatory agent, strongly inhibit the maturation and functioning of DCs via soluble inhibitory mediators [26].

We assumed that immunomodulatory properties of MSCs could be partially accomplished by regulation of TLRs expressions and functions. Therefore, we evaluated the expression levels of TLR3 and TLR9 transcripts in mouse DCs treated with MSCs conditioned media.

Our findings indicated that MSCs supernatant, harvested at 24–72-hr intervals, could significantly down-regulate TLR3 and TLR9 expressions at the mRNA level in DCs. In keeping with our results, a recent study has shown that MSCs inhibit TLR4 expression and signaling as well as DCs downstream molecular events, which impairs T cell priming and activation [15]. Several reports have demonstrated that many factors change TLR 2, 3, and 4 expression and signaling pathways [27-29]. However, the effect of MSCs on endosomal TLRs including TLR 3, 7, and 9 remained unidentified. Further investigations are needed to find the exact soluble mediators of MSCs responsible for TLR down-regulation of DCs.

Down-regulation of TLR9 on DCs causes immature DCs not to differentiate into mature DCs; they represent tolerogenic DCs with suppressive effects on immune response [30, 31]. Likewise, TLR3 down-regulated DCs produce ineffective amount of pro-inflammatory cytokines and fail to initiate antigen-specific inflammatory responses. 

In conclusion, MSCs may induce tolerogenic DCs phenotype by down-regulating TLR3 and TLR9 that culminate in ineffective adaptive immunity. Further analyses are needed to explore potential inhibition of mitogen-activated protein kinase and nuclear factor-κB activation as a downstream of TLRs for a better understanding of how DCs maturation and activation are regulated by MSCs. A functional study is also demanded to link TLR3 and TLR9 down-regulation to the inability of priming and activating T cells and mounting an effective antigen-specific immune response. Although the possible application of MSCs to treat immune-mediated disorders is currently under scrutiny, our findings confirm MSCs profound inhibitory effect on TLRs in DCs. Therefore, it may be considered a novel molecular inhibitory mechanism by which MSCs modulate DCs activation and maturation.
